# Green Phosphorene as a Promising Biosensor for Detection of Furan and p-Xylene as Biomarkers of Disease: A DFT Study

**DOI:** 10.3390/s22093178

**Published:** 2022-04-21

**Authors:** Aref Aasi, Erfan Aasi, Sadegh Mehdi Aghaei, Balaji Panchapakesan

**Affiliations:** 1 Small Systems Laboratory, Department of Mechanical Engineering, Worcester Polytechnic Institute, Worcester, MA 01609, USA; sagh0203@fb.com (S.M.A.); bpanchapakesan@wpi.edu (B.P.); 2 Department of Mechanical Engineering, Boston University, Boston, MA 02215, USA; eaasi@bu.edu

**Keywords:** prostate cancer, green phosphorene, sensor, DFT study, cancer biomarker

## Abstract

In this work, Green Phosphorene (GP) monolayers are studied as an electronic sensing element for detecting prostate cancer biomarkers from human urine. The adsorption of furan, C_8_H_10_ (p-xylene), and H_2_O on pristine GP and S- and Si-doped GP are investigated using the density functional theory (DFT) calculation. Furan and C_8_H_10_ molecules have been considered as important biomarkers of prostate cancer patients. First-principles DFT calculations are applied, and the results divulged that pristine GP could be a promising candidate for furan and C_8_H_10_ detection. It is manifested that furan and C_8_H_10_ are physisorbed on the S-, and Si-doped GP with small adsorption energy and negligible charge transfer. However, the calculations disclose that furan and C_8_H_10_ are chemically adsorbed on the pristine GP with adsorption energy of −0.73, and −1.46 eV, respectively. Moreover, we observe that a large charge is transferred from furan to the pristine GP with amount of −0.106 e. Additionally, pristine GP shows short recovery time of 1.81 s at room temperature under the visible light, which make it a reusable sensor device. Overall, our findings propose that the pristine GP sensor is a remarkable candidate for sensing of furan and other biomarkers of prostate cancer in the urine of patients.

## 1. Introduction

The analysis of urine enables the observation of biochemical processes and metabolic products in the human body, as a non-invasive method for screening disease states [1,2,3]. Human urine is composed of different molecules and analytes, and Volatile Organic Compounds (VOCs) are a fraction of the molecules among them. The odor signature of urine is produced by VOC substances, which carry information on physiological and metabolic status [4,5,6].

Today, over 279 kinds of VOCs (Furan, p-Xylene, aldehydes, ketones, etc.) have been identified in human urine that can be used as “urine-marks” to provide vital information about dysfunction or metabolic disorders in the human body [7,8]. Detection of VOCs in urine is a significant indicator for monitoring health conditions; hence, developing a suitable platform for this purpose is necessary [9,10,11].

Many studies have reported that canines can be trained to detect breast, lung, and ovarian cancers from breath, and urine samples [12,13,14]. It has been shown that variations in the concentration of VOCs from the breath and urine are potentially correlated with various types of diseases and cancers [15,16,17]. In this regard, scientists have demonstrated that urinary VOC patterns in cancer patients are often different from those found in the urine samples of control subjects, and these differences depend on cancer type and stage [18,19,20]. One of the leading types of cancer among men is prostate cancer, and furan and p-Xylene (C_8_H_10_) are reported biomarkers for this cancer [21,22]. While 5-year survival rates are nearly 100% for localized and regional prostate cancers, they are only 30% for distant prostate cancer. Sensors that could detect VOCs from breath and urine could indeed reduce the mortality rate for prostate cancers.

Semiconductor sensor technology based on nanomaterials is one of the latest methods in the field of urine analysis and detection of VOCs [21,23,24,25]. Since the introduction of graphene as the first 2D material, nanomaterial-based sensors have provided new opportunities in different fields, due to their unique properties [26,27]. Recently, Green Phosphorene (GP), as a novel 2D material and new allotrope of black phosphorene, has been theoretically proposed, and it has enticed considerable attention because of its exceptional characteristics of energy stability, tunable direct bandgap, and strong anisotropy. These properties have made it a great candidate in electronic, optical, catalysis, and sensing applications [28,29].

There have been several studies on the sensing capabilities of black phosphorene in the literature, but there are few theoretical studies regarding the molecule sensing application of GP. It offers faster electronic transport at room temperature and higher directional electronic anisotropy in comparison with the black phosphorene [30]. Inspired by these advantages, Mao et al. [31] theoretically investigated the sensitivity of GP toward inorganic compounds, such as NH_3_, SO_2_, HCN, and O_3_ molecules. They showed that GP could be a potential candidate for O_3_ detection. Kaewmaraya et al. [32] utilized theoretical DFT-based calculations to study interaction of GP with small molecules. They explored CO, CO_2_, NH_3_, NO, NO_2_, and H_2_O as major environmental molecules. In another study, [33], the interaction of GP with ethanol and methanol vapors based on theoretical DFT calculations are investigated. They suggested that GP nanosheet could be used as a platform to detect the existence of methanol and ethanol vapors. Very recently, our group theoretically examined the sensing properties of GP toward dissolved gases in oil transformers such as H_2_, CH_4_, and C_2_H_2_ and the findings manifested that GP could be used for the detection of these molecules [34].

Motivated by the fascinating structural and electronic properties of GP, in this paper, the interaction of GP with furan and p-Xylene, along with H_2_O as main interfering molecule in the urine are investigated. We utilize GP monolayer for detection of the targeted molecules, based on the first-principles method (DFT). Different structures of GP, such as pristine and doped GP, are systematically researched and we scrutinize the adsorption behaviors of the molecules with the GP-based nanosensor. Our results demonstrate the promising future of GP-based sensors in the development of high-performance room temperature VOC analyzers for detection of prostate cancer.

## 2. Computational Details

During this work, all DFT calculations were performed employing Atomistix ToolKit (ATK) package, associated with Non-Equilibrium Green’s Function (NEGF) [35,36,37].

The exchange-correlation functional was approximated using the Perdew–Burke–Ernzerhof (PBE) formalism. Additionally, the Van der Waals (VdW) and long-range interactions were considered by adopting Grimme (DFT-D2) algorithm. The adsorption of the molecules upon pristine or doped GP was found by adsorption energy, where the adsorption energy was calculated by:

(1) $$E_{ad}=E_{Doped-GreenP+molecule}-(E_{Doped-GreenP}+E_{molecule})$$

where $E_{Doped-GreenP+molecule}$, $E_{Doped-GreenP}$, and $E_{molecule}$ represent the energies for the pristine or doped GP-molecule system, pristine or doped GP, and the corresponding single molecule, respectively. The negative adsorption energy means that the process is exothermic, and its most negative value indicates that the adsorption process is energetically favorable. It is generally believed that chemisorption occurs when the absolute value of adsorption energy is greater than 0.8 eV [38,39].

The optimized lattice parameters of the bulk of GP were determined to be 10.62, 3.26, 7.93 Å along x, y, and z directions, respectively, and are in reasonable accordance with previous studies [32,33]. The supercell extended with a vacuum space size of 20 Å along the z-direction is considered to prevent image–image interactions. All the modeling was implemented on the sufficient supercell GP with dimensions of 13.82 × 13.25 Å^2^.

The basis set of Fritz-Haber-Institute (FHI) pseudopotentials with double-ζ polarized was employed for the calculations. In these calculations, 45 Hartree was set for the kinetic energy mesh cut-off. The convergence criteria of maximum force and maximum stress were set to 0.01 eV/Å and 0.001 eV/Å^3^, respectively. For sampling of the Brillouin zone, Monkhorst–Pack k-point was set as 20 × 20 × 1. The electronic properties of the configurations are investigated by the analysis of DOS and a 21 × 21 × 1 k-point was used.

Moreover, using Hartwigsen–Goedecker–Hutter (HGH) pseudopotentials with Tier 3 basis set, the charge transfer (Q) after adsorption of the molecule on the pristine or doped-GP sheet was calculated by employing Mulliken population analysis, where a negative Q demonstrates a charge conveyed from the molecule to the GP sheet, whereas a positive Q shows that the molecule extracts electrons from the sheet.

## 3. Results and Discussions

**Figure 1:**
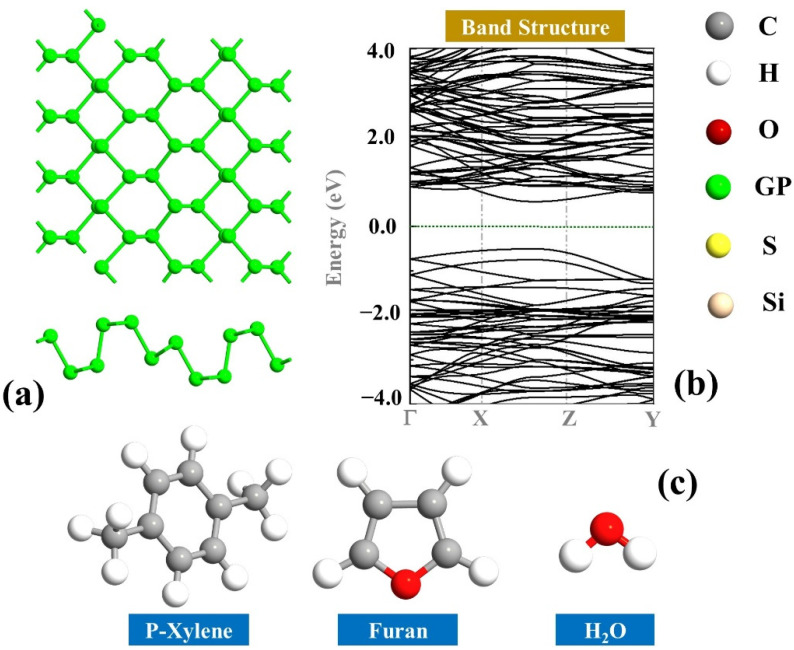
Illustration of (**a**) monolayer GP (top and side views), (**b**) band structure of the GP, and (**c**) structures of p-Xylene (C_8_H_10_), furan, and H_2_O molecules.

First, the optimized geometries of the molecules and the GP monolayer were obtained, and the results are depicted in Figure 1. It can be observed that the monolayer GP is a semiconductor and has an energy band gap of 1.06 eV [40,41,42]. Additionally, the optimized geometry of the considered molecules furan, C_8_H_10_ (p-Xylene), and H_2_O are shown in Figure 1. The full relaxation of the geometry of isolated furan gives C:C~1.37 Å, C:H~1.10 Å, and C:O~1.36 Å, which is in accordance with the experimental data [43]. Moreover, the optimized geometry of isolated C_8_H_10_ gives C:C~1.40 Å (on the benzene ring), C:H~1.10 Å, and C:C~1.51 Å (on the two sides), which is in good agreement with previous studies [44].

**Figure 2:**
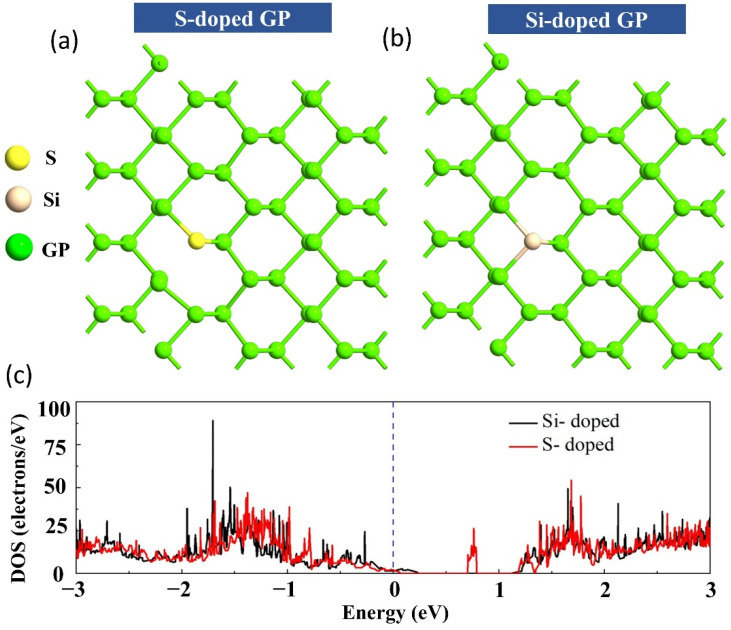
The relaxed atomic structures of (**a**) S-doped and (**b**) Si-doped GP sheet, along with (**c**) DOS calculations.

It is well known that nanomaterials such as GP prepared by the available fabrication methods are likely to have many defects. Additionally, they could be deliberately or accidentally doped with elements like S, and Si. Next, the interactions of S, and Si dopant upon GP were investigated. Two dopants (S, and Si) were introduced to the host material’s (GP) surface [32], and different potential points were examined; the most energetically optimized configurations are demonstrated in Figure 2.

Interestingly, it can be seen from Figure 2 that after doping the GP with the S atom, the structure underwent a distortion due to the comparable atomic sizes of the dopants with the phosphorus atom, while the bond of S with one of the adjacent P atoms was deteriorated.

Doping considerably impacts DOS, particularly the states close to the Fermi level. The DOS values for two dopant cases are shown in Figure 2. These changes are justified by the fact that GP possesses the sp^3^ hybrid character, and each P atom consists of a non-bonding lone pair of electrons that may engage with the frontier p-states of the dopants, and the S and Si doping cause the GP to convert to n-, and p-type semiconductors, respectively.

With the aim of obtaining the preferential adsorption sites of the molecules on the pristine GP, the molecules were placed at a distance of 2 Å above different locations of the sheet with diverse molecular orientations. Various adsorption sites on the GP surface, such as above the P hexagon, the P-P bond, and above the P atom, were studied.

**Figure 3:**
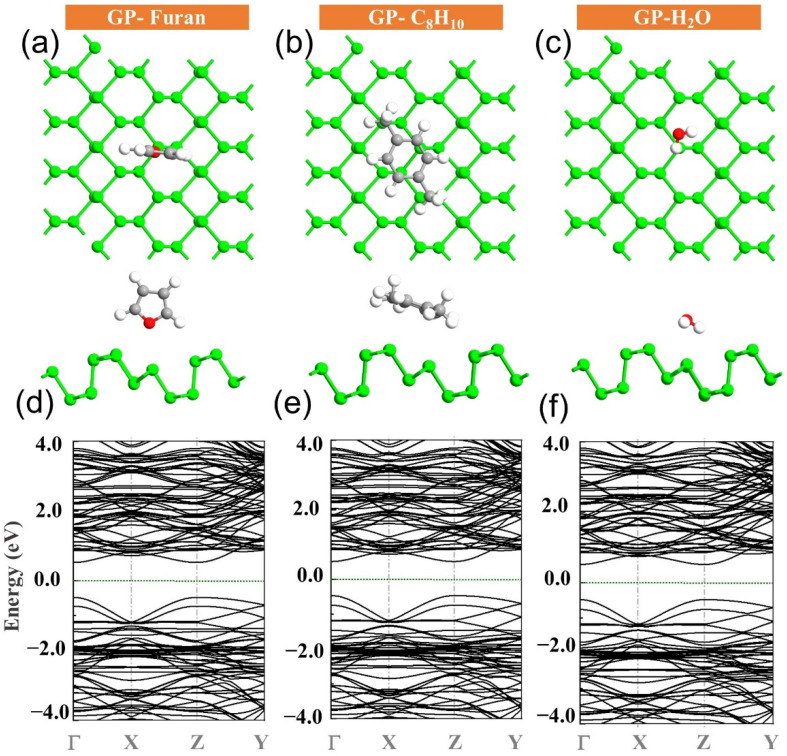
The most stable structures of (**a**) GP-furan, (**b**) GP-C_8_H_10_, and (**c**) GP-H_2_O with their corresponding band structures (**d**–**f**).

**Figure 4:**
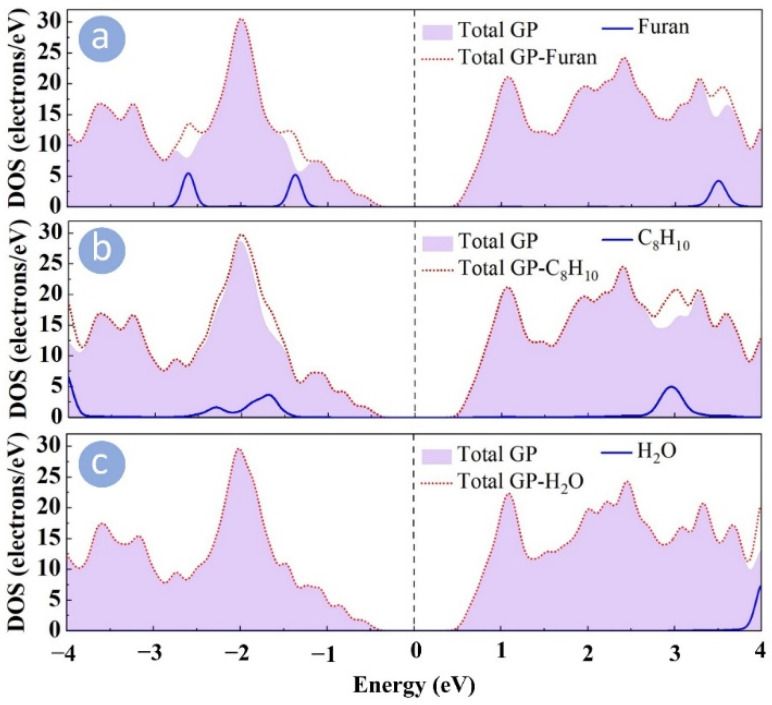
DOS plots for pristine GP sheet exposed to (**a**) furan, (**b**) C_8_H_10_, and (**c**) H_2_O molecules.

The interaction strength between the sensing material and analytes was evaluated by calculating the adsorption energies, and the most optimized structures are presented in Figure 3. Upon exposure of furan, it adopts a vertical direction, where its O atom is on top of the P atom with a minimum distance of 3.07 Å and an adsorption energy of −0.73 eV (Figure 4). From the electronic band structure calculations (Figure 3), it can be observed that the energy bandgap of the GP (1.06 eV) upon furan adsorption changes to 1 eV.

Moreover, after interaction of the GP with C_8_H_10_, it was adsorbed in a tilted parallel orientation with respect to the plane, with a minimum distance (H-P) of 3.09 Å. Energy of −1.46 eV is emitted after adsorption, and the energy bandgap changes to 0.978 eV. Finally, H_2_O is preferentially adsorbed in this manner, tilted from the horizontal orientation with respect to the GP surface and with the minimum distance of (H-P) 2.92 Å.

The adsorption energy value for H_2_O upon interaction with GP, as shown in Table 1, was found to be −0.5 eV. From the electronic band structure, it was determined that the energy bandgap changed to 0.997, and 1 eV, respectively. In addition, all the molecules provided electrons to the surface, so that a total net charge of 0.106 e was achieved for furan. Additionally, GP accepted a total net charge of 0.073 and 0.055 e from C_8_H_10_ and H_2_O molecules, respectively.

All of this information, such as net charge transfer and interaction distances for different systems, is detailed in Table 1. To gain a better understanding of molecule adsorption on the GP, the density of states (DOS) of the GP sheet was plotted along with different molecules in Figure 4.

It can be seen that the DOS of the H_2_O molecule disappears at around the Fermi level, which indicates that the molecule does not alter the electronic properties of GP, supporting the weak interaction between them. However, in the case of furan and C_8_H_10_, there are overlap peaks between the molecules and the GP, and the nearest peaks to the vicinity of the Fermi level are within the energy spans of −1.5 to −1 eV and −2 to −1.5 eV for furan and C_8_H_10_ adsorption, respectively. 

To track the adsorption mechanism and to shed light on the adsorption of the molecules upon the GP, recovery time (τ), as a critical factor for the evaluation of the sensor, has to be studied [25,45]. The recovery time τ captures the time cost for the desorption of a target molecule from the sensing material’s surface. The τ could be obtained according to the transition state theory and Van’t Hoff–Arrhenius explanation:

(2) τ=A−1exp(−EadBT)

where *A* represents the apparent frequency factor, *T* is the working temperature, and *B* is constant of Boltzmann (8.318 × 10^−3^ kJ/(mol·K)). The ambient temperature (300 K) is considered to gain a full understanding of the desorption properties of the sensor system. The frequency factor was determined to be 10^12^ and 10^16^ Hz, under visible and UV light conditions, respectively [46,47]. Parameter τ for different conditions is tabulated in Table 1.

From Equation (2), it can be observed that sensor devices with a lower value of τ were associated with lower *E*_ads_ at a given temperature. Thus, the recovery time τ at room temperature (300 K) and under visible light was determined to be 1.81, 3.3 × 10^12^, and 2.45 × 10^−4^ s for furan, C_8_H_10_, and water, respectively. It is important to highlight that both values of τ that are too long and too short are unfavorable for detection in real experiments [47]. As a result, GP is considered to be an option for the detection of furan molecules.

**Figure 5:**
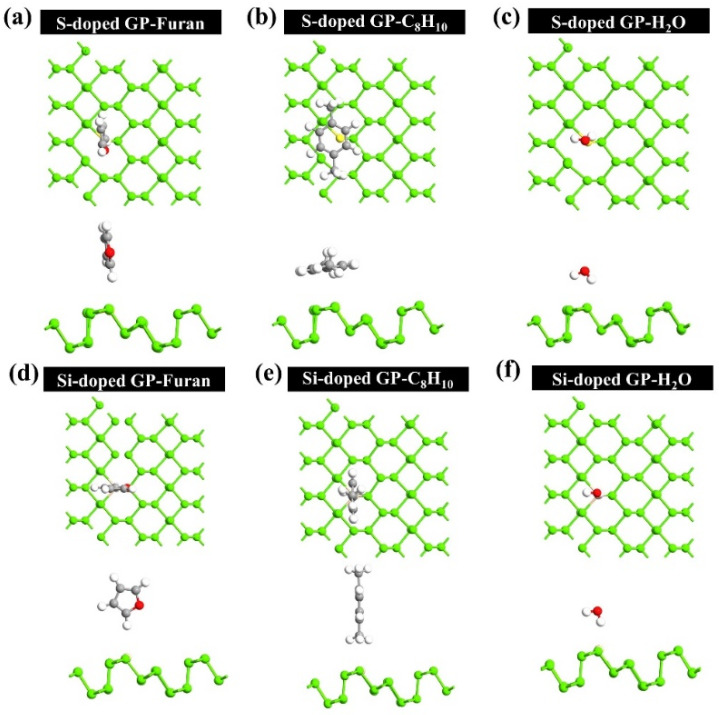
The most relaxed structures for adsorption of molecules on the S-doped and Si-doped GP surface. Configurations of S-doped (**a**) furan (**b**) C_8_H_10_ (**c**) H_2_O, and Si-doped (**d**) furan (**e**) C_8_H_10_ (**f**) H_2_O.

Subsequently, the optimized preferential adsorption configurations for the molecules on the S-, and Si-doped GP were investigated, and the results displayed in Figure 5. After adsorption of furan with S- and Si-doped GP, it was observed that furan adopts a vertical orientation with its H atom pointed toward the surface. There was a minimum distance of 2.59, and 2.82 Å between the H atom of furan and the S and Si atoms, respectively.

Furthermore, in the case of H_2_O on both S-, and Si-doped GP, it is adsorbed approximately vertically with respect to the surface of the monolayer, in which there is a minimum distance of 2.53, and 2.42 Å between the H atom and S, and Si atoms, respectively. Nonetheless, C_8_H_10_ displays a more complicated adsorption mechanism than other molecules, in such a way that it tends to be adsorbed on the S-doped GP horizontally, with a minimum distance of (H-S) 3.5 Å, and upon Si-doped GP, it is preferentially adsorbed vertically, where its H atoms face down to the Si atom and the surface with a minimum distance of 3.04 Å.

By comprehensively comparing the values of adsorption energy for the configurations, final adsorption energies of −0.07, −1.05, and −0.31 eV were obtained for the adsorption of furan, C_8_H_10_, and H_2_O on the S-doped GP, respectively. Additionally, E_ad_ values of −0.31, −0.33, and −0.16 eV were obtained for furan, C_8_H_10_, and H_2_O upon Si-doped GP, respectively.

**Figure 6:**
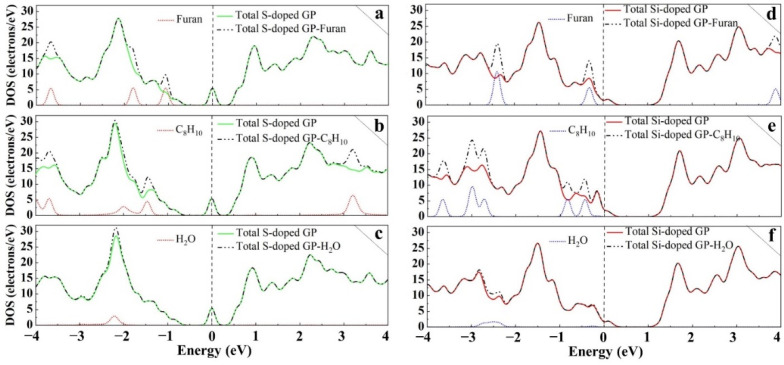
Total DOS curves for S- and Si-doped GP interaction with the molecules. Configurations of S-doped (**a**) furan (**b**) C_8_H_10_ (**c**) H_2_O, and Si-doped (**d**) furan (**e**) C_8_H_10_ (**f**) H_2_O.

DOS analysis was performed on the doped GP molecules structures, as shown in Figure 6, to further help reveal the nature of the interaction between the molecules and the structures. It can be seen that after adsorption of the molecules upon S- and Si-doped GP, there were no changes around the Fermi level, which suggests that the molecules did not alter the electronic properties of the substrate, supporting the weak interaction between them and the S- and Si-doped sheet.

Considering results obtained for the S- and Si-doped GP, all the molecules were weakly adsorbed onto the doped GP systems. When calculating the recovery time for the corresponding configurations, it can be observed that τ is either too short or too long for desorption of the molecules.

Eventually, the obtained results suggest that pristine GP is a promising factor in molecule sensing. Conversely, the findings show that by introducing atoms (S, and Si) to the GP, its sensitivity toward the prostate biomarkers is not improved, and there is low adsorption energy and negligible charge transfer between the doped GP and the molecules. However, pristine GP appears to be a good candidate for capturing furan and C_8_H_10_, with a moderate adsorption energy of −0.73, and −1.46 eV, large charge transfer, and a quick recovery time.

## 4. Conclusions

In brief, we employed first-principles computations to analyze the adsorption geometry, adsorption energy, charge transfer, and electronic band structure of GP with the adsorption of several molecules (furan, C_8_H_10_, and H_2_O). The results showed that pristine GP could be deployed as a base substrate for adsorbing prostate cancer biomarkers, such as furan and C_8_H_10_. While furan and C_8_H_10_ molecules were weakly adsorbed onto the surface of S-, and Si-doped GP, the results indicated that the adsorption energy was low and the charge transfer trivial. The adsorption energy for furan and C_8_H_10_ detection by pristine GP was determined to be −0.73 and −1.46 eV, respectively. Additionally, there was high transfer of charges, in an amount of 0.106 and 0.073 e, donated by furan and C_8_H_10_ to the GP’s surface. Furthermore, it was found that at room temperature and under visible light, pristine GP had a quick recovery time of 1.81 s, making it a reusable sensor for the detection of biomarkers. It is worth mentioning that biological probes are typically water solutions, and the content of water is superior. Therefore, chemical potential of molecules all competing for adsorption sites is concentration dependent. All in all, this study supports GP as a prominent adsorbing substrate for the diagnosis of prostate cancer biomarkers from exhaled breath, and deserves further attention.

## Figures and Tables

**Table 1:** The calculated adsorption energy (E_ad_), interaction distance (D)—i.e., the distance between the molecule and the GP sheet, charge transfer (Q), where negative values of charge indicate a charge transfer from the molecule to the surface, and recovery time (τ).

System	E_ad_ (eV)	D (Å)	Q (*e*)	τ sec @ T = 300 K (Visible Light)	τ sec @ T = 300 K (UV Light)
Pristine GP	-	-	-	-	-
Pristine GP-Furan	−0.73	3.07	−0.106	1.81	1.81 × 10^−4^
Pristine GP-C_8_H_10_	−1.46	3.09	−0.073	3.3 × 10^12^	3.3 × 10^8^
Pristine GP-H_2_O	−0.5	2.92	−0.055	2.45 × 10^−4^	2.45 × 10^−8^
S-doped-GP	-	-	-	-	-
S-doped-GP-Furan	−0.07	2.59	0.027	1.49 × 10^−11^	1.49 × 10^−15^
S-doped-GP-C_8_H_10_	−1.05	3.50	−0.083	4.3 × 10^5^	43.02
S-doped-GP-H_2_O	−0.31	2.53	0.003	1.6 × 10^−7^	1.6 × 10^−11^
Si-doped-GP	-	-	-	-	-
Si-doped-GP-Furan	−0.31	2.82	0.007	1.6 × 10^−7^	1.6 × 10^−11^
Si-doped-GP-C_8_H_10_	−0.33	3.04	−0.03	3.48 × 10^−7^	3.48 × 10^−11^
Si-doped-GP-H_2_O	−0.16	2.42	0.03	4.86 × 10^−10^	4.86 × 10^−14^

## Data Availability

The data presented in this study are available on request from the corresponding author.
